# Temperature control in critically ill patients with a novel esophageal cooling device: a case series

**DOI:** 10.1186/s12871-015-0133-6

**Published:** 2015-10-19

**Authors:** Ahmed F. Hegazy, Danielle M. Lapierre, Ron Butler, Eyad Althenayan

**Affiliations:** 1 Department of Anesthesia and Perioperative Medicine, University of Western Ontario, London Health Sciences Centre, University Hospital, 339 Windermere Road, London, N6A 5A5 ON Canada; 2 Department of Medicine, Division of Critical Care, University of Western Ontario, London Health Sciences Centre, University Hospital, 339 Windermere Road, London, N6A 5A5 ON Canada

## Abstract

**Background:**

Mild hypothermia and fever control have been shown to improve neurological outcomes post cardiac arrest. Common methods to induce hypothermia include body surface cooling and intravascular cooling; however, a new approach using an esophageal cooling catheter has recently become available.

**Methods:**

We report the first three cases of temperature control using an esophageal cooling device (ECD). The ECD was placed in a similar fashion to orogastric tubes. Temperature reduction was achieved by connecting the ECD to a commercially available external heat exchange unit (Blanketrol Hyperthermia – Hypothermia System).

**Results:**

The first patient, a 54 year-old woman (86 kg) was admitted after resuscitation from an out-of-hospital non-shockable cardiac arrest. Shortly after admission, she mounted a fever peaking at 38.3 °C despite administration of cold intravenous saline and application of cooling blankets. ECD utilization resulted in a temperature reduction to 35.7 °C over a period of 4 h. She subsequently recovered and was discharged home at day 23. The second patient, a 59 year-old man (73 kg), was admitted after successful resuscitation from a protracted out-of hospital cardiac arrest. His initial temperature was 35 °C, but slowly increased to 35.8 °C despite applying a cooling blanket and ice packs. The ECD was inserted and a temperature reduction to 34.8 °C was achieved within 3 h. The patient expired on day 3. The third patient, a 47 year-old man (95 kg) presented with a refractory fever secondary to necrotizing pneumonia in the postoperative period after coronary artery bypass grafting. His fever persisted despite empiric antibiotics, antipyretics, cooling blankets, and ice packs. ECD insertion resulted in a decrease in temperature from 39.5 to 36.5 °C in less than 5 h. He eventually made a favorable recovery and was discharged home after 59 days. In all 3 patients, device placement occurred in under 3 min and ease-of-use was reported as excellent by nursing staff and physicians.

**Conclusions:**

The esophageal cooling device was found to be an effective temperature control modality in this small case series of critically ill patients. Preliminary data presented in this report needs to be confirmed in large randomized controlled trials comparing its efficacy and safety to standard temperature control modalities.

## Background

Uncontrolled fevers have been associated with poor neurological outcomes in various critically ill patient populations. Post arrest anoxic brain injury, ischemic and hemorrhagic strokes, and traumatic brain injury patients all appear to be particularly sensitive to the detrimental effects of high body temperature [1, 2]. Most authorities would therefore recommend tight control of body temperature in these settings [3–8].

Hypothermia is commonly induced using a combination of internal and external cooling modalities. Internal cooling modalities include intravenous administration of cold crystalloids and intravascular cooling catheters [9]. External or body surface cooling can be achieved using cooling blankets, adhesive pads, and ice packs [10]. Each of these methods however, has its limitations [11, 12].

Administration of intravenous refrigerated crystalloid (4 °C) boluses is a simple, effective and widely available method of hypothermia induction [13]. Lack of precise temperature control and the potential for pulmonary edema however, make this modality unsuitable for the maintenance phase of hypothermia [14, 15]. Surface cooling methods, such as cooling blankets and ice packs, often cause shivering, skin breakdown, and in obese patients may be ineffective [9]. Intravascular cooling catheters require the time of a physician for insertion and bear the potential risks of line infection and deep venous thrombosis [11, 12]. Searching for a temperature control modality that overcomes the limitations, inefficiencies and inconveniences of the existing methods is therefore strongly desired.

The ideal temperature control modality should be effective, safe, and easy to use. An esophageal cooling device has recently become available which may theoretically possess some of these attributes. We hereby report the first use of this novel temperature control device in a series of three critically ill patients.

## Methods

**Fig. 1:**
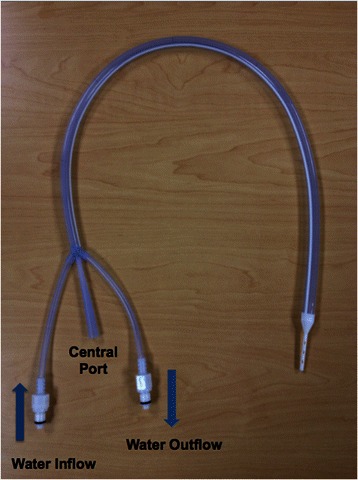
Esophageal cooling device. Esophageal cooling device showing water inflow/outflow tubes and central (gastric) port

The Esophageal Cooling Device**®** (Advanced Cooling Therapy, Chicago, IL) is a cooling/warming device that connects to standard heat exchange units. By design, it is a multi-lumen, disposable, silicone tube (Fig. 1), placed in the esophagus with its tip situated in the stomach. A central inner lumen allows gastric drainage, while cold or warm water circulate within an outer circumferential cavity. Esophageal Cooling Device (ECD) models compatible with the Blanketrol Hyper-Hypothermia System**®** (Cincinnati Sub-Zero, Cincinatti, OH) and the Medi-Therm Hyper/Hypothermia System**®** (Stryker, Kalamazoo, MI) are commercially available.

Prior to ECD utilization, written informed consent was obtained from the patients’ substitute decision makers. Consent was also obtained to use patient information for publication. Communication with the Health Sciences Research Ethics Board of Western University revealed that a board review prior to consent and publication is not required. ECD’s are approved for clinical use by Health Canada in addition to our local institutional regulatory bodies.

ECD placement technique was similar to that of standard orogastric tubes. After the appropriate depth of placement was determined externally, the tip and proximal shaft were lubricated with a water-soluble lubricant. The ECD was then connected to the water-circulating heat exchange unit, which was turned “on” allowing water to flow through the device. This made the ECD stiffer facilitating subsequent esophageal advancement [16, 17]. Correct placement was then confirmed by chest X-ray.

As per the manufacturer’s recommendations, the central (gastric) port was connected to a low level of intermittent suction after initial ECD placement. The ECD product monograph currently states that the gastric port is to be used for suction only and should not be used for enteral feeding. Communication with the manufacturer revealed that the reason for this restriction was that an application for approval was not sought to use this port for feeds. After confirming adequate placement in a gastric location, the authors felt that the benefits of using the gastric port for enteral access outweigh the potential risks. The central port was therefore subsequently used for enteral medication administration and trophic feeds in all three patients off-label, uneventfully.

All patients were monitored with temperature-sensing urinary catheters. The heat exchange unit (Blanketrol Hyper-Hypothermia System) was set on “Auto Control” mode. With this modality, the temperature of water circulating through the system is automatically adjusted by the heat exchange unit in order to match a chosen setpoint. The operator in charge of regulating the target temperature was the ICU nurse. Both bladder and target temperatures were recorded hourly.

Our current institutional protocol for comatose post cardiac arrest survivors aims at maintaining patient temperatures in a range of 34 to 36 °C for a period of 24 h. The induction protocol comprises the administration of 1 litre boluses of refrigerated normal saline (at 4 °C), up to a maximum total of 4 litres. Additionally, our protocol allows for the application of ice packs to the groin and axilla, and a cooling blanket (Maxi-Therm® Lite Blankets, Cincinnati Sub-Zero, Cincinatti, OH) directly to the patients skin during the induction and maintenance phases of hypothermia. All patients receive intravenous infusions of an opioid (fentanyl or hydromorphone) and a hypnotic (midazolam or popofol) during the 24 h of hypothermia. Use of neuromuscular blocking agents is left at the discretion of the treating physician who may choose to reserve it’s use for shivering treatment only. After 24 h of hypothermia, passive rewarming is initially attempted. Active rewarming with a warming blanket is only used if the patient does not rewarm passively within 6 h. Use of the ECD enabled induction, maintenance and rewarming of our first two patients. In our third patient, the ECD was used primarily as a fever control modality.

## Results

### Patient A

**Fig. 2:**
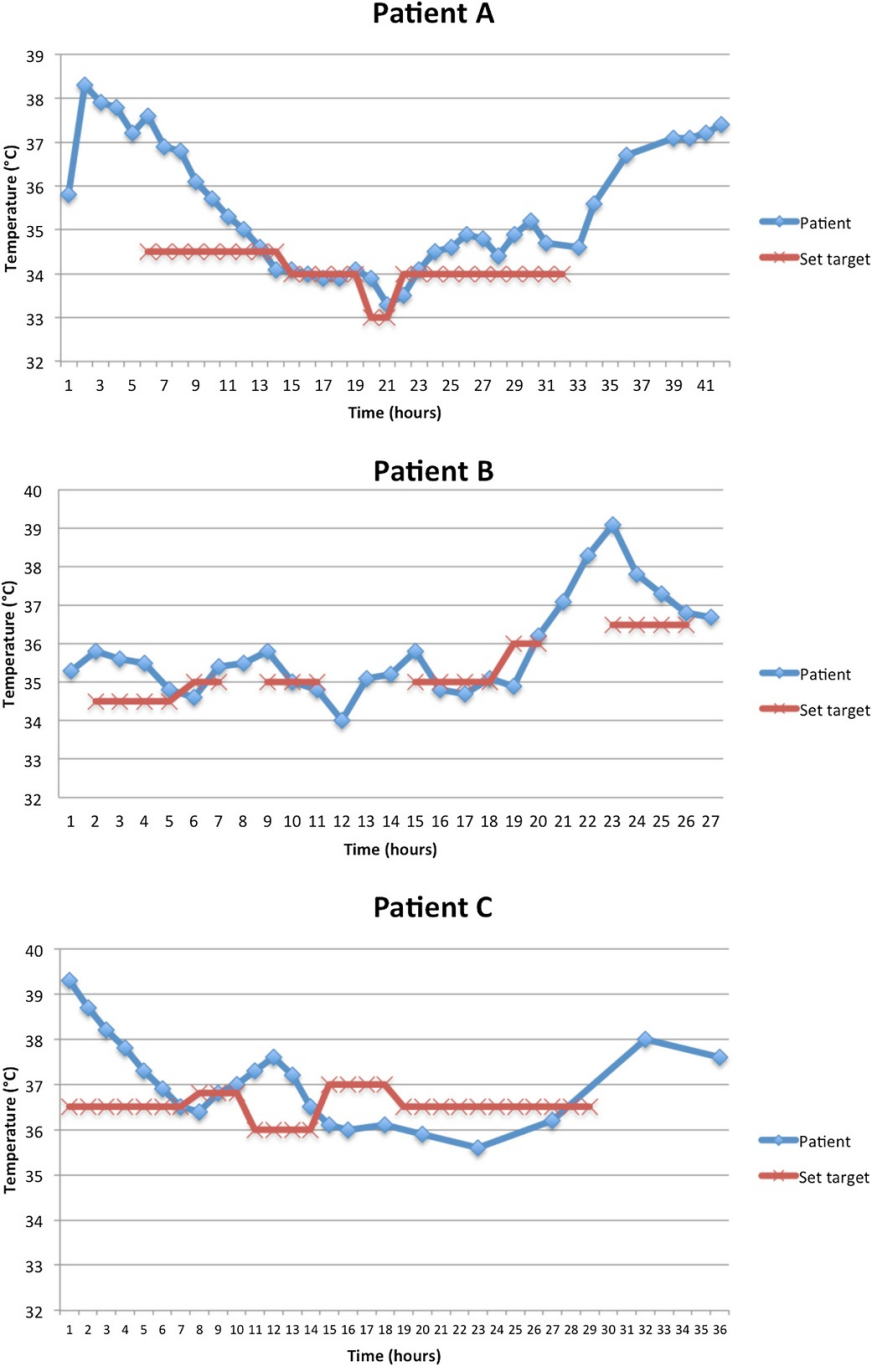
Patient temperatures after esophageal cooling device insertion in comparison to set targets. Top: Patient A. Middle: Patient B. Bottom: Patient C

A 54 year-old woman (86 kg, BMI 30.1) was transferred to the critical care unit after successful resuscitation from an out-of-hospital pulseless electrical activity (PEA) cardiac arrest. Her past medical history was significant for severe chronic obstructive pulmonary disease requiring home oxygen. Post resuscitation, she remained comatose and a seizure episode was witnessed in the ICU. Her temperature rapidly increased to 38.3 **°**C one hour after admission, upon which a cooling blanket was applied and 2 litres of intravenous cold saline were administered. These interventions resulted in only a mild reduction of temperature, reaching 37.6 **°**C after 4 h (Fig. 2 a). At that point, an ECD was placed to aid in temperature reduction.

After confirming adequate connection of the ECD to the external heat exchange unit, we were able to induce hypothermia despite the patient’s ongoing febrile response. Patient temperature was reduced, from 37.6 to 35 **°**C, within 6 h. During hypothermia induction, the cooling blanket was kept on. After induction, the cooling blanket was removed and hypothermia was maintained with the ECD alone. The bedside nurse adjusted the external unit’s temperature targets on more than one occasion. This was noted to quickly reflect on the patient’s body temperature (Fig. 2 a). After 26 h of ECD use, the device was removed.

Over the next few days, the patient showed signs of neurological recovery. Due to her pre-existing respiratory disease however, weaning from mechanical ventilation was prolonged. Extubation was achieved on day 16, after which she made a full recovery and was discharged home on day 23.

### Patient B

A 59 year-old man (73 kg, BMI 23.8) was admitted to the critical care unit after successful resuscitation from an out-of-hospital PEA cardiac arrest. Return of spontaneous circulation was obtained after 1 h from initiation of CPR. Post resuscitation, the patient exhibited signs of cardiogenic shock and pulmonary edema. ST elevation in the lateral leads of his ECG led to an urgent coronary angiogram. This revealed no significant coronary artery disease but severe mitral regurgitation was found. An intra-aortic balloon pump was inserted and he was transferred to the critical care unit on both epinephrine and norepinephrine infusions.

His initial temperature on intensive care admission was 35 **°**C. No temperature interventions at that time were therefore required. Approximately 7 h later, his temperature slowly began to rise, despite the use of a cooling blanket and the application of ice packs to his axilla and groin. At that time, the patient's severe hypoxia due to acute pulmonary edema, precluded the use of intravenous refrigerated crystalloids for temperature reduction. The ECD was therefore inserted for temperature modulation for the remaining 17 h.

Despite the patient’s rising temperature trajectory, a reduction of temperature from 35.8 to 34.8 **°**C was achieved within 3 h using the ECD (Fig. 2 b). Shivering was observed and an intravenous bolus of cisatracurium was given. The bedside nurse was allowed to adjust the external unit’s temperature targets at her discretion to achieve the desired patient temperature. On multiple occasions however, the nurse turned the unit off after the patient had reached target temperature assuming it was no longer needed. After completing 24 h of hypothermia, rewarming from 34.9 to 36.2 **°**C was achieved within 2 h using the ECD. The unit was then turned off. Over the next few hours the patient spiked a fever peaking at 39.1 **°**C, upon which the heat exchange unit was turned on again and set to a target of 36.5 **°**C. Over the next 3 h, the patient’s temperature steadily approached target, coming down to 36.8 **°**C (Fig. 2 b). Unfortunately, the patient continued to show signs of poor neurological prognosis during his critical care stay. A clinical diagnosis of post-anoxic brain death was made on day 3.

### Patient C

A 47 year-old man (95 kg, BMI 34.9) was admitted to the intensive care unit for postoperative care after an emergent coronary artery bypass grafting procedure. Preoperatively, he had experienced symptoms suggestive of a respiratory tract infection with dyspnea, high-grade fevers and leukocytosis. In the postoperative period, gradual deterioration of his respiratory status was observed despite empiric antibiotic therapy. On postoperative day 6, he developed an acute respiratory distress syndrome and a CT scan of the thorax revealed evidence of multifocal necrotizing pneumonia. An increasing temperature refractory to broad-spectrum antibiotic therapy, regular acetaminophen administration, cooling blankets, and application of ice-packs to the groin and axilla triggered the insertion of the ECD for fever control. His temperature at the time was uncontrolled, peaking at 39.3 **°**C.

After insertion of the ECD, a temperature reduction from 39.3 to 36.9 **°**C was achieved within 4 h. Over the next few hours, the patient’s temperature fluctuated (Fig. 2 c), leading the nurse to change the set target accordingly. During the whole time the ECD was in place, a cisatracurium infusion was ongoing for optimization of mechanical ventilation. No evaluation of the device as a potential trigger of shivering is therefore available for this patient. His hospital stay was remarkable for sepsis-induced multi-organ failure in addition to atrial and ventricular arrhythmias that were observed since his admission. After a long stay in hospital, he eventually recovered and was discharged home on postoperative day 59.

No complications directly attributable to the ECD were observed for the three reported cases. The ECD required less than three minutes for its placement to be successfully completed. Ease-of-use was described as excellent by both nurses and physicians.

## Discussion

Our report is the first to describe the successful use of an esophageal cooling device in humans. Insertion of this device was found to reliably decrease body temperature of critically ill patients refractory to standard cooling modalities. On average, temperature reduction occurred at a rate of 0.52 **°**C/hr. This rate was significantly lower than that reported in previous animal studies utilizing the ECD [18–20].

Kulstad et al. were the first to report esophageal cooling in an animal study involving swine as large as 70 kg in weight [18]. All swine were anesthetized with isoflurane and endotracheally intubated. ECD utilization resulted in an average cooling rate of 1.2 **°**C/hr. After 36 h of continuous use, no histologic evidence of esophageal mucosal damage was found. In addition, no shivering was observed in any of their anesthetized swine during ECD use. In our series however, shivering was observed in one patient (patient B) in the absence of a cooling blanket. Shivering is a very common side effect of surface cooling techniques [21] and its avoidance is highly desirable. Morbid effects of shivering include an increase in myocardial oxygen consumption, lactic acidosis, and a resistance to cooling, all of which can be deleterious in the post cardiac arrest patient [22].

Anatomically, the esophagus is situated posterior to the heart and major vessels. The ECD’s ability to modulate body temperature is therefore likely secondary to heat transfer with the central circulation. The slower cooling rate in our patients compared to previous animal reports, might be due to multiple factors. Interspecies differences are likely implicated. In addition, our patients appeared to be actively mounting a fever at the time of ECD utilization. Lastly, postcardiac arrest patients commonly have myocardial stunning and a low cardiac output. This would likely result in a slower circulation impairing systemic cooling. The average cooling rate observed with the ECD in this series was however, comparable to recently published cooling rates of other more commonly used cooling devices. Endovascular cooling devices have been recently reported to have an average cooling rate of 0.39 **°**C/hr, while surface cooling devices were reported to have an average cooling rate of 0.27 **°**C/hr [23].

This method of cooling offers several advantages over the currently available modalities. The esophageal-cooling device can be easily and quickly inserted upon patient arrival to the ICU. Its small size does not interfere with bedside interventions (e.g. line insertions, coronary angiograms) and diagnostic studies. This is sharply contrasted to the use of intravascular cooling catheters and cooling blankets. Intravascular cooling catheters offer precise temperature control but their insertion may be a source of time delay [24]. Cooling blankets are generally patient-encompassing appliances that can obstruct access to the patient for provision of care. Consequently their application is frequently delayed until other necessary interventions have been completed. Rapid infusion of ice-cold saline is another alternative modality that is frequently effective, but lacks precise temperature control [12]. In addition, administration of large amounts of fluids may be necessary to lower body temperature. This bears the potential of inducing fluid overload and pulmonary edema in certain at-risk patients. And lastly, the central mechanism of cooling provided by the ECD may prove useful for cooling of obese patients whose body habitus makes conventional surface cooling techniques less effective [25].

Utilization of the esophageal environment for heat transfer comes with significant limitations. The ECD is currently approved for use for up to 36 h only, which limits the duration of temperature control this technique can provide. In addition, esophageal exposure to intraluminal cold temperatures has been known to cause a reduction in esophageal motility [26–29]. Further studies would be required to evaluate if this reduction in esophageal motility is associated with morbidity (such as aspiration and/or infection). Contraindications to ECD use include patients with known esophageal deformity, evidence of esophageal trauma, known ingestion of acidic or caustic poisons within the prior 24 h and patients less than 40 kg in weight. Although a potentially very useful tool, further data is needed to characterize the patients that would benefit most from ECD use, compare it with other temperature control modalities and delineate the possible associated adverse events.

## Conclusions

The esophageal cooling device was found to be an effective temperature control modality in this small case series of critically ill patients. Preliminary data presented in this report needs to be confirmed in large randomized controlled trials comparing its efficacy and safety to standard temperature control modalities.

### Key messages

- The esophageal cooling device (ECD) appears to be an effective temperature control modality that can be used for both cooling and rewarming.
- The average rate of cooling in this case series was 0.52 **°**C/hr.
- Shivering was observed in one out of the three patients that were cooled with the ECD.
- Insertion of the device required less than 3 min and ease-of-use was described as excellent by nursing staff and physicians alike.

## Abbreviations

BMI: body mass index
CT: computerized tomography
ECD: esophageal cooling device
ECG: electrocardiogram
hr: hour
ICU: intensive care unit
kg: kilogram
PEA: pulseless electrical activity
